# Computed Tomography-Based Angiographic Evaluation of Graft Patency Rate After Coronary Artery Bypass Graft Surgery in Bangladesh

**DOI:** 10.7759/cureus.28902

**Published:** 2022-09-07

**Authors:** Redoy Ranjan, Kevin Matthew Sales, Asit Baran Adhikary

**Affiliations:** 1 Cardiac Surgery, Bangabandhu Sheikh Mujib Medical University, Dhaka, BGD; 2 Cardiac Surgery, St. George's University Hospitals NHS Foundation Trust, London, GBR

**Keywords:** endarterectomy, coronary artery bypass graft, graft patency, ct angiogram, cabg

## Abstract

Background

This study aims to assess the graft patency rate following coronary artery bypass graft (CABG) surgery using noninvasive CT angiography.

Materials and methods

A total of 68 patients were retrospectively evaluated with CT angiography (group I: 34 patients with coronary endarterectomy (CE) and group II: 34 patients without CE). CE was performed in multi-segmental diffuse coronary artery disease (CAD) or when calcified or extremely thick plaques made anastomosis troublesome. A team of two experts, an interventional radiologist and a cardiac surgeon, did the evaluations of graft patency rate.

Results

A total of 205 bypass grafts were evaluated in 68 post-CABG status patients (110 grafts in group I and 95 grafts in group II; moreover, 82 were arterial and 123 were venous grafts). Post CABG, CT angiography demonstrated a graft patency rate of about 90% in both study groups at five years follow up, which was statistically insignificant (P > 0.05) in terms of graft patency rate. Following CE, five-year angina-free survival rates were 89% and 91% in groups I and II, respectively.

Conclusion

CABG surgery with endarterectomy is reliable and effective. It achieves the desired surgical myocardial revascularization in patients with diffuse calcified CAD having no alternative options for adequate myocardial revascularization.

## Introduction

Atherosclerosis is the most common form of arteriosclerosis and a complex multifocal progressive inflammatory arterial disease of medium and large-size arteries involving multiple genetic and environmental factors [1,2]. The most common cardiovascular disease, cause of death in middle-aged and older patients in developing countries, and the number one cause of death in the western world is atherosclerosis of the coronary artery [3,4]. Depending on the symptoms, risk stratification, or risk and pattern of coronary artery disease (CAD), the mode of treatment may be either medication, percutaneous coronary intervention (angioplasty/stenting), or coronary artery bypass surgery (CABG) [5,6].

According to the American Heart Association (AHA), CABG surgery is the most common intervention in patients with ischemic heart disease (IHD). Techniques of CABG surgery can be divided into two categories: traditional on-pump CABG and off-pump CABG (OPCABG) surgery [4-8]. Currently, IHD patients present with complex and diffused CAD, with several comorbidities that make complete surgical revascularization more challenging [2,5-9]. In 1957, coronary endarterectomy (CE) was first performed as a surgical option for myocardial revascularization. The initial endarterectomies results observed a greater incidence of perioperative morbidity and mortality [2-5]. However, it is essential to perform an endarterectomy to anastomose severely calcified and diffusely diseased coronary arteries that are not suitable to perform adequate myocardial revascularization with only bypass grafting. The principal concept of CE is to extract the atheromatous plaque in complex CAD in order to achieve uninterrupted distal runoff in diseased coronary arteries [2-6]. However, the absence of authentic guidelines poses a challenge to many cardiac surgeons, making them reluctant to perform endarterectomies. The published papers from the last decades have shown that long-term outcomes, particularly survival rates, have improved in diffuse and calcified CAD, which have undergone CE [4,7,8]. Following a CE procedure, postoperative anticoagulation has been critical in preventing perioperative myocardial infarction. In recent studies, authors have demonstrated that strict postoperative protocols for anticoagulation management with early systemic heparin infusion bridging to antiplatelet and warfarin therapy provide improved graft patency rate [6-9].

Comparative studies on the long-term outcome of off-pump CABG with or without endarterectomy have not been done in Bangladesh. This study aimed to assess the long-term graft patency rate following OPCABG with endarterectomy, and provide details regarding triple anticoagulation (antiplatelet, warfarin) strategies for patients having CE due to diffuse calcified CAD.

## Materials and methods

A retrospective study was performed between September 2019 and February 2020, and 68 patients were evaluated in two study groups. Group 1 had 34 patients who had CE with OPCABG surgery; Group 2 had 34 patients who had only OPCABG surgery. This study included only postoperative isolated off-pump CABG with or without endarterectomy patients who attended outpatient department follow-up after five years of surgery. However, patients with combined CABG and valvular or congenital cardiac procedures, redo CABG, and systemic diseases such as end-stage renal disease, hepatic failure, and respiratory failure were excluded from the study. The primary study endpoint was to evaluate left ventricular function and long-term graft patency rates at a five-year follow-up. To evaluate the primary outcome, patients underwent transthoracic echocardiography (TTE) and CT-based coronary angiogram. However, the secondary study endpoints considered the incidence of major adverse cardiac and cerebrovascular events (MACCE), postoperative myocardial infarction, and transient ischemic attack (TIA)/stroke during the five-year clinical follow-up. The departmental, academic, and National Research Ethics Committee (NREC) have given ethical clearance for this study. Informed written consent was obtained from the patients involved. A quality assurance system was employed to confirm the validity of the observations and findings to ensure the reliability of the data and to conclude that it was derived correctly from the raw data. Quality control measures were applied to each stage of data handling to ensure data consistency and correct processing.

Indication and surgical technique of CE

The CE techniques have been published in detail elsewhere [7]. A conclusive decision to do endarterectomy was made per-operatively in the presence of diffuse coronary lesion or multi-segmental lesion without a good distal run-off, and when a calcified or extremely thick plaque burst, making anastomosis troublesome or hindering the distal stream. We performed close technique endarterectomy by slow sustain and continuous traction of atheromatous plaque with delicate Forceps. Furthermore, the overall arteriotomy incision was ~15 mm long, and the atheromatous plaque was carefully inspected for a smooth distal tapper end to ensure complete expulsion. The backflow of blood from the distal endarterectomy coronary artery is a consoling indication of adequate plaque removal.

Postoperative anticoagulation therapy

In this study, we have used triple anticoagulation therapy with a target international normalized ratio (INR) of 1.5-2.5, according to the local hospital protocols described elsewhere [5,7]. Triple therapy comprises initial systemic heparinization bridging to warfarin and a combination of clopidogrel and aspirin (75 mg) from the first postoperative day to the next six months in anticipation of acute graft thrombosis in the endarterectomies as well as a native coronary artery. Postoperative unfractionated heparin was used (usually 5000 IU; S/C dose eight hourly), administered ~3 hours following surgery if bleeding is minimum, and bridging to warfarin (~5 mg) until the third postoperative day. From the fourth postoperative day, 2.5-5 mg warfarin was used for the next ~6 months, and the dose was titrated according to the target INR of 1.5-2.5 level. 

Statistical analysis

Statistical analyses were performed utilizing SPSS v25.0 (IBM, Chicago, IL, USA). Data were expressed as mean ± SD for continuous variables, percentages, and frequency for categorical variables. Pearson’s chi-square evaluates categorical variables. The Kaplan-Meier curve estimates the cumulative survival rate. A p-value of ≤0.05 was considered significant.

## Results

A total of 68 post-CABG patients (group I: 34 OPCABG patients with endarterectomy; group II: 34 OPCABG patients without endarterectomy) underwent CT angiographic evaluation in this review after five years of operation. A total of 205 bypass grafts (110 bypass grafts in group I and 95 grafts in group II) were evaluated in this study. Moreover, the total number of arterial and venous grafts among the study population was 82 and 123, respectively. Table 1 shows sociodemographic and operative data of study populations. There were ~1.5 endarterectomies performed per patient, and about two-thirds of endarterectomy were from the left coronary territory. However, ~40% of the left anterior descending artery needed endarterectomy and was revascularized with the left internal thoracic artery. The mean grafts were 3.25±0.5 and 2.75±0.25 in groups I and II. Blood requirement was significantly higher in CE cohorts (p<0.05), but the mortality and other postoperative morbidity were similar between the two groups.

**Table 1:** Sociodemographic characteristics of study population (N=68). Note: Left Main stem Disease: >50% lesion in left main coronary artery; LVEF: Left ventricular ejection fraction; NYHA: New York Heart Association; CCS: Canadian Cardiovascular Society; EuroSCORE: European System for Cardiac Operative Risk Evaluation; IMA: Internal mammary artery; RSVG: Reverse saphenous venous graft; MI: Ayocardial infarction; DM: Diabetes mellitus; PCI: Percutaneous intervention; IHD: Ischemic heart disease. All study variables were statistically insignificant between study groups (P >0.05).

Variables	CE with CABG (n = 34)	Only CABG (n = 34)
Age (mean) in years	61.25 ± 2.5	59.50 ± 2.5
Male	27 (79.4%)	24 (70.6%)
Hypertension	29 (85.3%)	28 (82.4%)
Dyslipidemia	25 (73.5%)	26 (76.5%)
Smoking	26 (76.5%)	23 (67.6%)
Previous MI	25 (73.5%)	25 (73.5%)
Type 2 DM	22 (64.7%)	22 (64.8%)
Prior PCI	7(20.6%)	7(20.6%)
Family history of IHD	18 (52.9%)	19 (55.9%)
Left main stem disease	6 (17.6%)	5 (14.7%)
Original logistic EuroSCORE	5.8±1.7	5.8±1.6
CCS Class 3-4	23 (67.6%)	22 (64.7%)
NYHA class 3-4	22 (64.7%)	21 (61.8%)
Use of cardiopulmonary bypass	4 (11.8%)	2 (5.9%)
LVEF >50%	15 (44.1%)	21 (61.8%)
LVEF 30-50%	13 (38.3%)	9 (26.5%)
LVEF <30%	6 (17.6%)	3 (8.8%)
Mean number of grafts	3.25±0.5	2.75±0.25
Used conduits		
Left IMA	34 (30.9%)	29 (30.5%)
Right IMA	5 (4.6%)	2 (2.1%)
Radial artery	4 (3.6%)	8 (8.4%)
RSVG	67 (60.9%)	56 (58.9%)
Number of endarterectomized artery (N=45)		
Left anterior descending artery (LAD)	18 (40.0%)	-
Obtuse marginal artery	7 (15.5%)	-
Diagonal artery	5 (11.2%)	-
Right coronary artery (RCA)	8 (17.8%)	-
Posterior descending artery	3 (6.7%)	-
LAD + RCA	2 (4.4%)	-
LAD + Diagonal	2 (4.4%)	-

**Figure 1:**
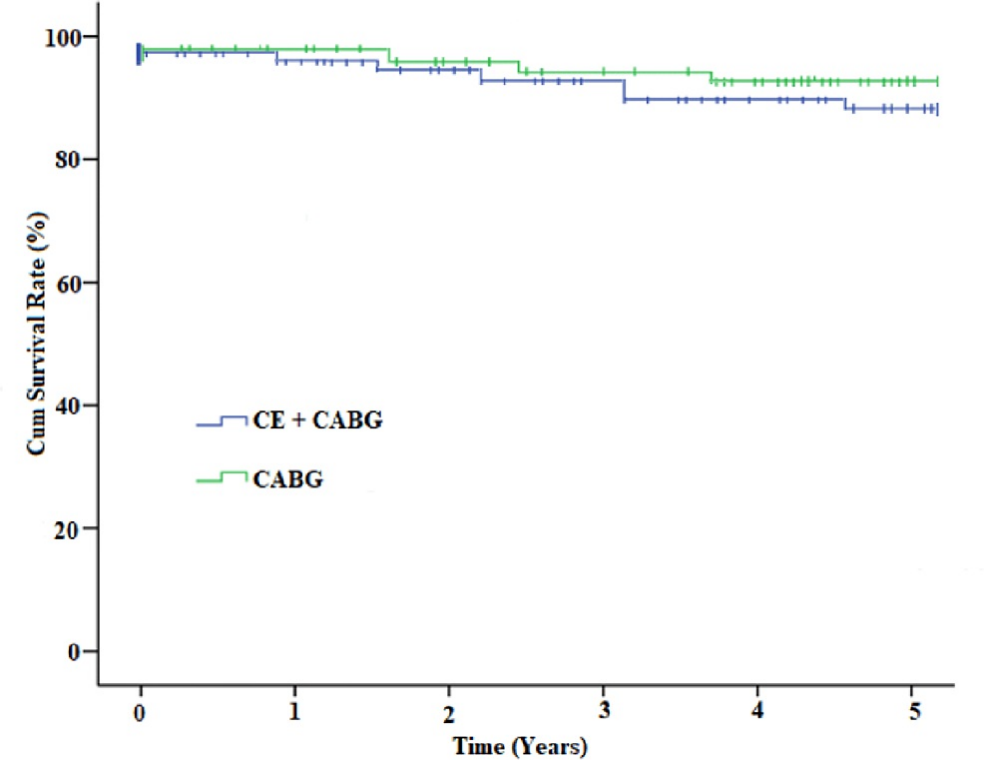
K-M survival curve observes the long-term freedom from angina of endarterectomy with OPCABG compared to isolated CABG. CABG: Coronary artery bypass graft; OPCABG: Off-pump coronary artery bypass graft; K-M survival curve: Kaplan-Meier survival curve.

Tables 2-3 demonstrate postoperative early and long-term outcomes following CABG surgery. At five years of follow-up, approximately 88% and 91% of patients were in New York Heart Association (NYHA) Class 1-2 (angina-free survival) in groups I and II, respectively. Furthermore, transthoracic echocardiography evaluation found a comparable prevalence of >50% left ventricle ejection fraction among ~62% and ~68% of patients in Groups I and II, respectively. At the five-year follow-up, the CT angiogram evaluated a total of 205 grafts from 68 post-CABG patients, and the graft patency rate was similar, about 90% in both study groups (Table 3). The Kaplan-Meier survival curve found that 89.5% and 91.0% of patients were free from angina in groups I and II at five years of follow-up, respectively (Figure 1).

**Table 2:** Early postoperative outcome variables of study population. P-value is calculated from Chi-squared test and p-value of ≤0.05 is considered as statistically significant. AF: Atrial fibrillation; MI: Myocardial infarction; TIA: Transient ischemic attack; IABP: Intra-aortic balloon pump.

Variables		CE with CABG (n=34)	Only CABG (n=34)	P-value
Hospital stays (days)		9 ± 1.5	9.5 ± 1	1.00
Post-operative AF		14.7%	10.66%	<0.001
Post-operative acute MI		5.9%	2.9%	<0.001
Renal failure		2.9%	5.9%	0.715
Respiratory failure		2.9%	2.9%	0.811
Neurological complications	TIA	5.9%	0.0%	0.002
	Psychosis	2.9%	2.9%	0.608
Postoperative blood transfusion (units)		1.75 ± 0.25	1.25 ± 0.5	<0.001
Postoperative hemorrhagic complications		5.9%	2.9%	<0.001

**Table 3:** Long-term postoperative outcome variables of study population. P-value is calculated from Chi-square test and p-value of ≤0.05 is considered as statistically significant. NYHA: New York Heart Association; LVEF: Left ventricle ejection fraction; CABG: Coronary artery bypass graft.

Variables	CE with CABG (n=34)	Only CABG (n=34)	P-value
Long-term outcome at five years follow-up			
Regular follow-up	31 (91.2%)	30 (88.2%)	0.209
NYHA functional class I-II	30 (88.2%)	31 (91.2%)	0.275
Transthoracic echocardiogram (TTE)			
LVEF >50%	21 (61.8%)	23 (67.7%)	<0.001
LVEF 30-50%	9 (26.5%)	10 (29.4%)	
LVEF <30%	4 (11.7%)	1 (2.9%)	
CT angiogram at five-year follow-up			
Number of graft (n=205)	N=110 Graft	N=95 Graft	0.640
Patent (<50% lesion)	99 (90.0%)	86 (90.5%)	
Stenosis (50-75% lesion)	8 (7.3%)	6 (6.3%)	
Occluded (>75% lesion)	3 (2.7%)	3 (3.2%)	

## Discussion

The patency of the bypass grafts and native coronary artery is vital in the long-term outcome after surgical myocardial revascularization. However, recently published papers observed that approximately 40% and 25% of post-CABG patients suffered from a recurrence of angina and graft restenosis at five-year follow-up, respectively [3-6]. Therefore, a noninvasive CT imaging modality is preferable in contrast to invasive conventional coronary angiography with substantial cost and procedure-related complications and mortality [5-7]. The newly established multi-slice CT scan employs multi-row detector array systems permitting a rapid imaging modality allowing views of cardiac structures during one breath hold; this allows scan times of up to 0.25s [7-10]. This study has shown that approximately 75% of endarterectomies performed on the left coronary territory have had ~2% and ~0.6% early mortality in ICU and 30-day mortality, respectively. However, mortality is commonly observed in a particular group of patients, those who have experienced left anterior descending artery (LAD) endarterectomy, multi-vessel endarterectomy, and pre-existing LV dysfunction (EF<30%), similar to other published studies articles [10-14]. Moreover, strict postoperative adherence to anticoagulation therapy is vital in preventing perioperative MI, following the CE procedure [15-19]. Several published articles have also evaluated the effectiveness of the systemic heparin infusion as bridging until warfarin has reached the target INR level of 2.0-2.5 [3,8,18-22].

However, Ghatanatti R and Teli A performed a meta-analysis of 43 recently published papers. They observed that endarterectomy with OPCABG surgery in diffuse calcified CAD has a better outcome [23]. It was seen that using an open endarterectomy technique provided better long-term graft patency rates; however, at the five-year follow-up, the survival curve remained static for both open- and closed-technique endarterectomy. Nonetheless, Qiu Z et al. said that CE is a safe and feasible technique with excellent mid-term survival and graft patency rates for a diffuse calcified group of CAD patients [24]. Nevertheless, Naseri E et al. found a higher incidence of restenosis in graft and native endarterectomies artery with ~7% postoperative MI rate, higher than current study findings [25].

In this current study, a CT angiogram observed a ~91% graft patency rate at five-year follow-ups, similar to other recent study results [5,8,21-24]. The better graft patency rate may be due to strict adherence to anticoagulation protocol by combining warfarin and dual antiplatelet agents. It is essential to consider that isolated CABG is insufficient to provide the desired myocardial revascularization in diffuse calcified CAD, presenting in-stent restenosis and poor LV function. Concomitant endarterectomy with CABG is needed to provide good distal run-off to achieve a better postoperative outcome [5-10,20-25]. However, postoperative triple anticoagulation therapy and surgical skills remain essential. Endarterectomy was not found to be an independent predictor of either poor long-term survival or graft patency rate in this study.

This current study has a few limitations, such as this retrospective study was conducted in a single center with a five-year follow-up period. It is quite likely that there are subtle differences in the cohort of the patients that would cause the surgeon to elect to perform an endarterectomy instead of a more conventional CABG procedure, leading to a selection bias. Furthermore, as this current study is based on outpatient department post-CABG patients, the plausibility of bias related to selective reporting and measurement of study outcomes cannot be excluded. Despite these limitations, there is no simple method to answer questions about how to increase grafts' long-term patency to endarterectomized vessels, and this study offers potentially valuable insights. A randomized control trial with a large population and long-term follow-up are necessary to confirm our findings and define both groups' long-term clinical and functional results. Developing a well-trained cardiac, surgical, and anesthetic team and establishing modern equipment and adequate logistic support should be done in the cardiac institutes of Bangladesh to ensure up-to-date cardiac services and research for complex CAD.

## Conclusions

This study observed a standard and comparable long-term graft patency rate of ~90% following OPCABG surgery with or without endarterectomy in Bangladesh. Therefore, as a surgical option, CE is a viable procedure for desired surgical myocardial revascularization in diffuse calcified CAD patients and has good long-term graft patency and survival benefits. However, endarterectomy should be considered an adjuvant therapy to CABG surgery in patients with diffusely calcified coronary arteries, even when no alternative could achieve complete myocardial revascularization.
